# Publisher Correction: A cdk1 gradient guides surface contraction waves in oocytes

**DOI:** 10.1038/s41467-017-02520-1

**Published:** 2018-01-10

**Authors:** Johanna Bischof, Christoph A. Brand, Kálmán Somogyi, Imre Májer, Sarah Thome, Masashi Mori, Ulrich S. Schwarz, Péter Lénárt

**Affiliations:** 1 Cell Biology and Biophysics Unit, European Molecular Biology Laboratory (EMBL), Meyerhofstrasse 1, 69117 Heidelberg, Germany; 2 Institute for Theoretical Physics and BioQuant, Heidelberg University, Philosophenweg 19, 69120 Heidelberg, Germany

Correction to: *Nature Communications* 10.1038/s41467-017-00979-6, published online 11 October 2017

An incorrect version of the Supplementary Information was inadvertently published with this article, where the wrong file was included. The HTML has been updated to include the correct version of the Supplementary Information.

