# Supplementary material for: A cdk1 gradient guides surface contraction waves in oocytes
Source: Nat Commun. 2017 Oct 11;8:849. doi: 10.1038/s41467-017-00979-6 (PMC5636809; doi:10.1038/s41467-017-00979-6)
Supplement: Supplementary file 1 — Supplementary Information [file 41467_2017_979_MOESM1_ESM.pdf]

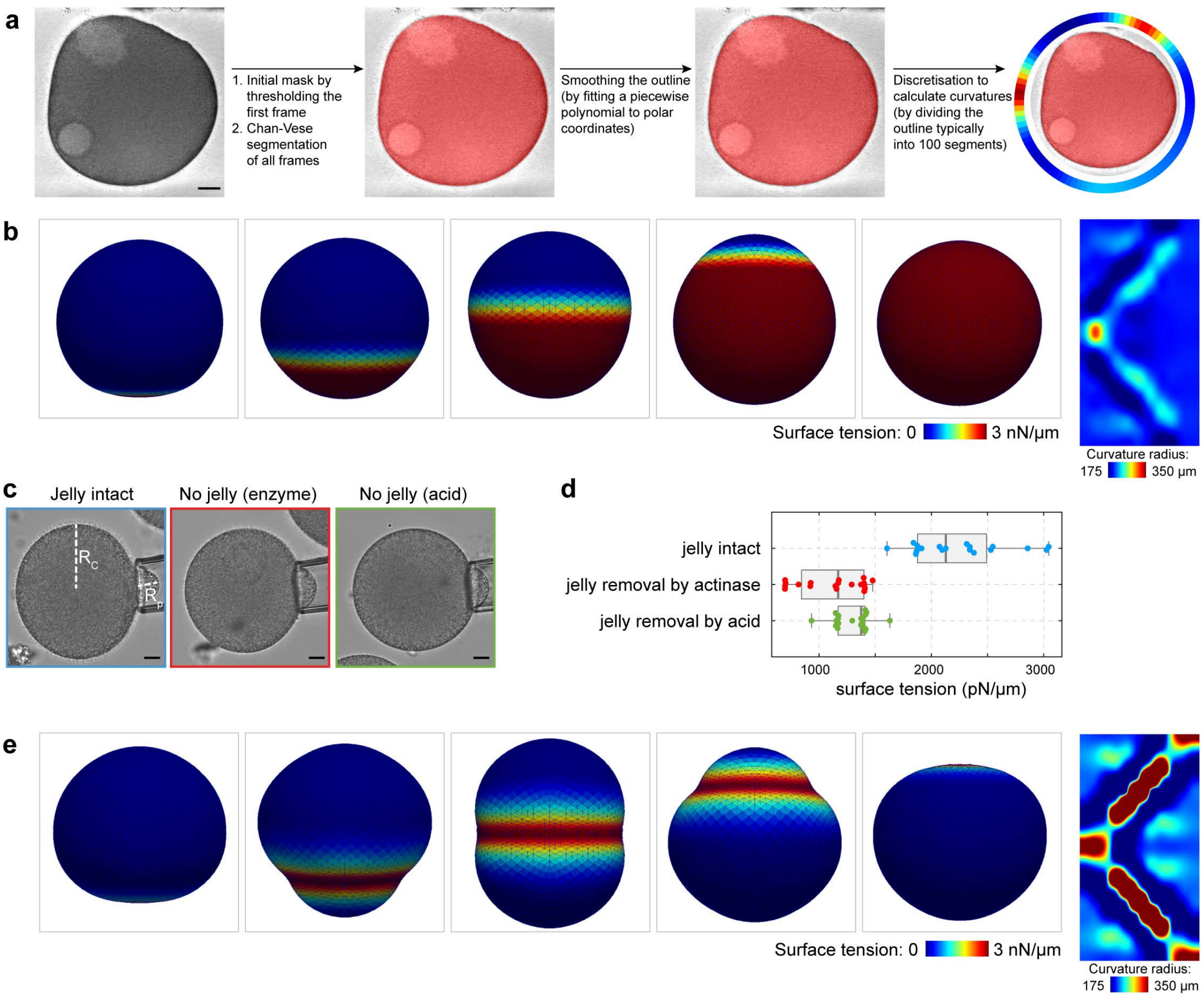

Supplementary Figure 1: Quantification and modelling of shape changes during SCWs **(a)** An example frame illustrating key steps of the segmentation and surface curvature calculation pipeline. **(b)** 3D mechanical model of an oocyte during the contraction wave with a single wave front, not limited to a band. Selected frames and respective kymograph of curvature radii are shown. Surface tension and radii of curvature values are encoded in the pseudo-colour scale shown on the bottom right. **(c)** Selected images of an oocyte with an intact jelly layer or with jelly layer removed either by enzymatic or acidic sea water treatment as indicated during surface tension measurement using pipette suction. **(d)** Removal of the jelly coat by either enzymatic (red) or acid (green) treatment leads to a softening of the oocytes compared to controls with intact jelly layers (blue). Dot plots of measurements of individual oocytes overlaid with box plots of the same data. **(e)** Selected frames of

3D mechanical model of the oocyte undergoing contraction with a lowered surface tension, recapitulating the effect of jelly removal.

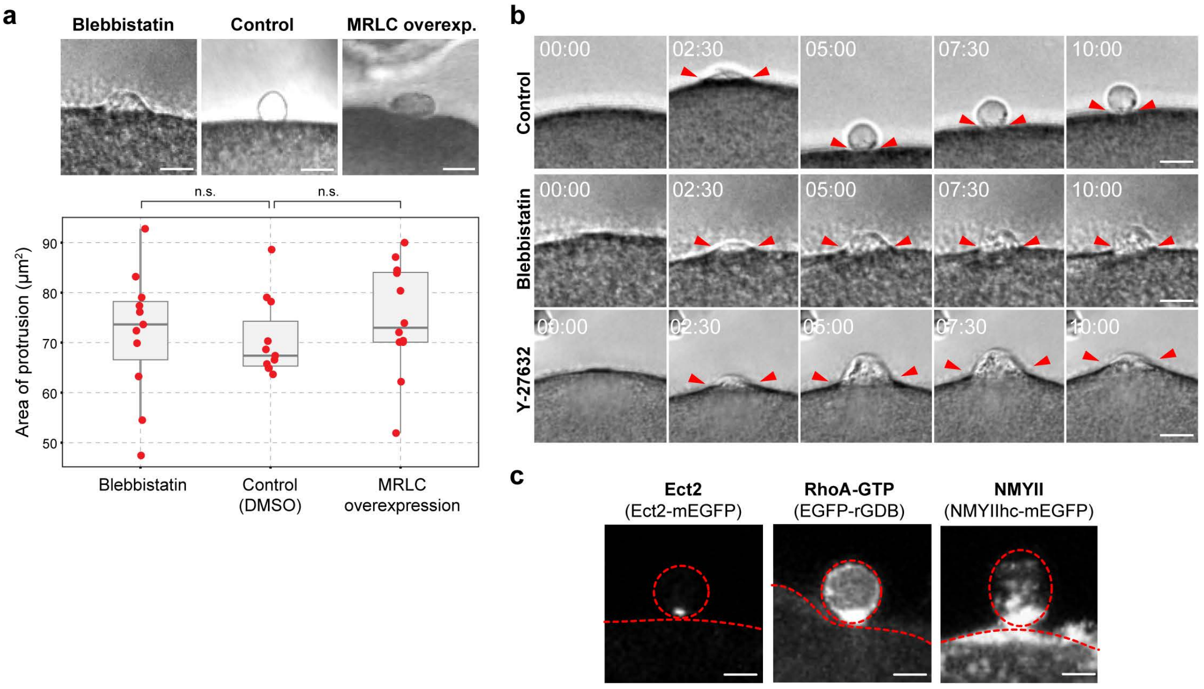

Supplementary Figure 2: The size of polar body protrusion is independent of SCW. (**a**) Still frames and quantifications of the area at the maximum size of protrusion of polar bodies in oocytes treated with 300 µm Blebbistatin, DMSO, and in oocytes overexpressing MRLC. Dot plots of measurements of individual oocytes overlaid with box plots of the same data. Scale bar 10 µm. n.s. not significant, determined via ANOVA. **(b)** Still frames of polar body formation in an untreated control oocyte, an oocyte treated with Blebbistatin or injected with the Rok inhibitor Y-27632, showing inhibition of polar body closure. Scale bars: 10 µm. Time in mm:ss. Red arrow heads indicate site of polar body extrusion. **(c)** Images of the components of the contractile ring at polar body closure showing localisation of Ect2, RhoA-GTP and NMYII. Scale bars: 10 µm. Red dashed line shows the outline of oocytes and polar bodies.

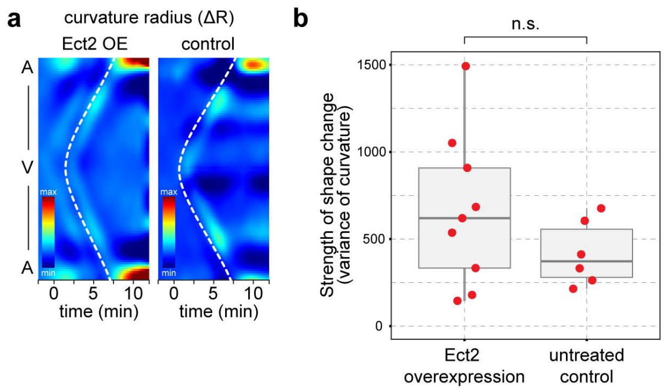

Supplementary Figure 3: Suggestive evidence for involvement of Ect2 in SCW. (a) Kymographs showing the shape changes associated with SCW in an Ect2 overexpressing and an untreated control oocyte. Radii of curvature values are encoded in the pseudo-colour scale bottom left. **(b)** Overexpression of Ect2 does increase variability but does not significantly affect the strength of the SCW as measured by the variance of curvature. Dot plots of measurements of individual oocytes overlaid with box plots of the same data. n.s. not significant, determined via ANOVA.

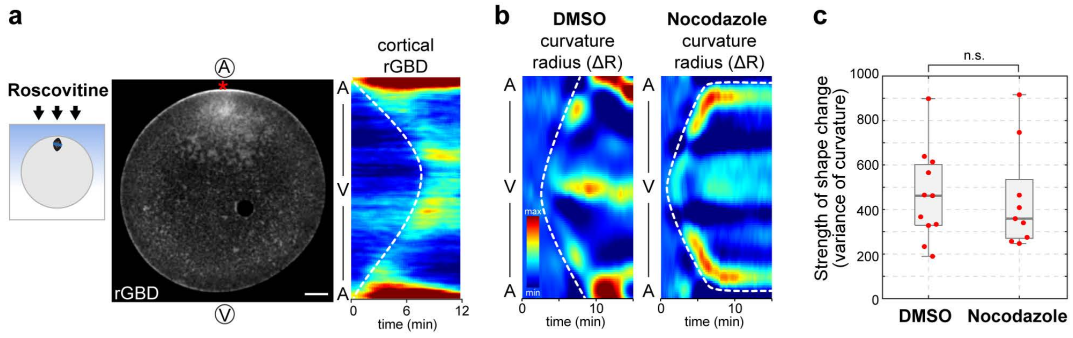

Supplementary Figure 4: SCW is regulated by cdk1 independently of microtubules **(a)** Single selected frame at the start of the SCW of an oocyte expressing RhoA-GTP marker EGFP-rGBD and locally treated with the cdk1 inhibitor Roscovitine (as marked on the scheme to the left). Kymograph of the cortical rGBD signal (as for Fig 3a) is shown on the right. Scale bar 20 µm. The starting point of the SCW is marked by the red asterisk. **(b)** Kymographs showing the change in curvature radii during the SCW in a control oocyte and an oocyte with microtubules removed by Nocodazole. Radii of

curvature values are encoded in the pseudo-colour scale inset. **(c)** Quantification of the strength of the shape change during the SCW in control oocytes treated with DMSO and in oocytes treated with Nocodazole. Dot plots of measurements of individual oocytes overlaid with box plots of the same data. n.s. not significant, determined via ANOVA.

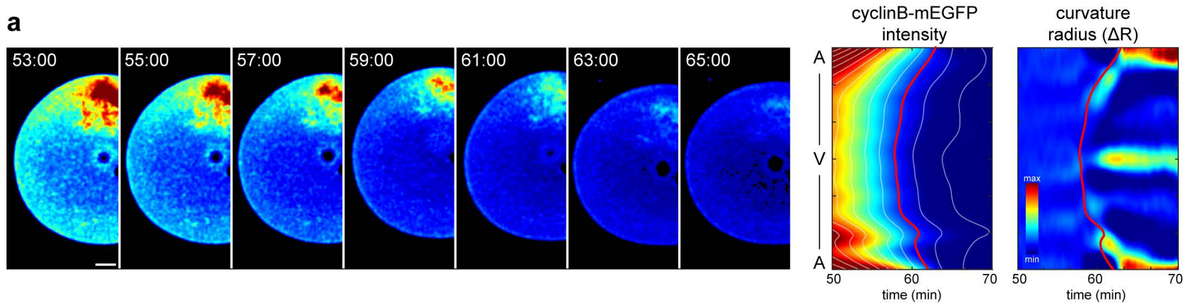

Supplementary Figure 5: Overexpression of cyclinB alters the cdk1-cyclinB gradient and thus SCW pattern **(a)** Selected pseudo-coloured frames of a time-lapse recording of an oocyte overexpressing cyclinB-EGFP. Kymographs of subcortical cyclinB-EGFP fluorescence intensity with isolines in white. In red, highlighted isoline conforming to the SCW, which is overlaid onto the second kymograph to the right showing the radii of curvature during the SCW in this oocyte. Radii of curvature values are encoded in the pseudo-colour scale inset. Scale bar 20 µm. Time in mm:ss.

Supplementary Table 1. *Parameters used in the 3D surface mechanics simulations*

Bending stiffness	κ_b	$2 \cdot 10^{-4}\, nN\mu m$
Peak surface tension	σ	$3\, nN/\mu m$
Stretch modulus	K_α	$5\, nN/\mu m,$<br>$2.5\, nN/\mu m$ without jelly
Shear modulus	μ	$K_\alpha/2$
Volume constant	k_V	$10^{-2}\, nN/\mu m^2$

Supplementary Table 2. *Parameters used in the simulation of the cdk1-cyclinB reaction-diffusion system*

Cell radius	R	$90\, \mu m$
Nucleus radius	r	$40\, \mu m$
Distance Nucleus-AP	d	$5\, \mu m$
Diffusion constant Cdk1	D_{cdk1}	$87\, \mu m^2/min$
Diffusion constant APC/C	D_{APC}	$348\, \mu m^2/min$
Initial Cdk1 concentration in the nucleus	$c_{0,n}$	$2500\, \mu m^{-3}$<br>$(4.2\, \mu M)$
Initial Cdk1 concentration in the cytoplasm	$c_{0,c}$	$500\, \mu m^{-3}$<br>$(0.83\, \mu M)$
Degradation rate Cdk1	k_0	$8.7 * 10^{-3} min^{-1}$
APC/C-dependent Cdk1 degradation rate	k_1	$0.87 \mu m^{-3} min^{-1}$<br>$(1.5\, nM\, min^{-1})$
Cdk1 production rate in the nuclear region	k_2	$34.8 \mu m^{-3} min^{-1}$<br>$(59\, nM\, min^{-1})$
Degradation rate APC/C	k_3	$8.7 * 10^{-3} min^{-1}$
Cdk1-dependend APC/C production rate	k_4	$0.0053\, min^{-1}$
EC50 Cdk1 degradation	J	$10 \mu m^{-3}\ (17\, nM)$
EC50 APC/C degradation	K	$10 \mu m^{-3}\ (17\, nM)$
