# Supplementary material for: A cdk1 gradient guides surface contraction waves in oocytes
Source: Nat Commun. 2017 Oct 11;8:849. doi: 10.1038/s41467-017-00979-6 (PMC5636809; doi:10.1038/s41467-017-00979-6)
Supplement: Supplementary file 3 — Description of Additional Supplementary Files [file 41467_2017_979_MOESM3_ESM.pdf]

Description of Additional Supplementary Files

File Name: Supplementary Information
Description:

File Name: Peer Review File

File Name: Supplementary Movie 1
Description: **Supplementary Movie 1 (related to Fig 1b):** Overview of the SCW in starfish oocytes.
Transmitted light images overlaid by channels labelling microtubules (red, EB3-mEGFP3) and chromosomes (green, mCherry-H2B). Frame is 220x220 µm, time is as mm:ss.

File Name: Supplementary Movie 2
Description: **Supplementary Movie 2 (related to Fig 2a):** Localization of NMYII during meiosis I. NMYIIhcmEGFP is imaged in an equatorial plane across the animal-vegetal axis. Frame is 238x238 µm,
time is as mm:ss.

File Name: Supplementary Movie 3
Description: **Supplementary Movie 3 (related to Fig 2g):** RhoA activity during meiosis visualised by the RhoA-GTP marker EGFP-rGBD. Frame is 241x241 µm, time is as mm:ss.

File Name: Supplementary Movie 4
Description: **Supplementary Movie 4 (related to Fig 2h):** RhoA activity during meiosis visualised by the RhoA-GTP marker EGFP-rGBD in an oocyte injected with the Rok inhibitor Y-27632. Frame
is 241x241 µm, time is as mm:ss.

File Name: Supplementary Movie 5
Description: **Supplementary Movie 5 (related to Fig 3d):** Spatiotemporal dynamics of cyclinB-EGFP
during meiosis in starfish oocytes. cyclinB-mEGFP is imaged in an equatorial plane across the animal-vegetal axis. Frames are 15 sec averages of a high-speed recording (1.5 s/frame). The movie plays twice, with contrast adjusted to visualize the gradient at SCW for the second time.
